# Parental play beliefs as a factor in the frequency of preschoolers’ requests for parent–child play and its duration

**DOI:** 10.3389/fpsyg.2026.1749925

**Published:** 2026-05-21

**Authors:** Margarita Gavrilova, Elena Dvorskaya

**Affiliations:** 1 Department of Educational Psychology and Pedagogy, Faculty of Psychology, Lomonosov Moscow State University, Moscow, Russia; 2 Laboratory of Childhood Psychology and Digital Socialization, Federal Scientific Center for Psychological and Interdisciplinary Research, Moscow, Russia

**Keywords:** family factors, parental beliefs, parental retrospective childhood experience, parent–child play, play beliefs, preschool age

## Abstract

Parent–child play is broadly acknowledged to be a crucial context for child development within the cultural-historical approach. However, the associations between family factors—parental play beliefs, family socio-demographic characteristics, and the parental retrospective childhood experiences—and indicators of parent–child play remain an understudied area. This study aimed to examine associations between these family factors and such parent-reported indicators of parent–child play as the frequency of child’s requests for parent–child play and its duration. The study involved 557 parents (M_age_ = 37.0, SD = 5.3), predominantly mothers (94%), of children attending preparatory preschool groups in Russia (M_age_ = 6.0, SD = 0.3; 46% girls), corresponding to the final year before school entry. Parents completed the Parent Play Beliefs Scale (PPBS) and reported on the frequency of child’s requests for parent–child play, its duration, and their own retrospective childhood experiences. The analysis revealed that parental Play Support was positively associated with the frequency of child’s requests and the duration of parent–child play, while Academic Focus showed no significant relationship with either play indicator. Moreover, parental age and education were indirectly associated with the frequency of the child’s requests through Play Support, which is consistent with indirect-only mediation. The results suggest that the frequency of child’s requests is associated with parental beliefs about the developmental value of play rather than directly with their age or education. Within the exploratory aim of the study, parental retrospective childhood experiences were associated with parental play beliefs: Play Support was negatively associated with frequent moving, whereas Academic Focus was positively associated with an unstructured home environment. The findings may be relevant for educational programs aimed at helping parents recognize the developmental value of play and support parent–child play in everyday family contexts.

## 1 Introduction

From the perspective of the cultural-historical approach, parent–child play is considered a crucial condition for the gradual transition to the mature form of play (Vygotsky, 2017; Elkonin, 1978). Despite the recognized developmental potential of play, contemporary research indicates that parents often prioritize structured learning activities from an early age (Oshchepkova et al., 2023; Plotnikova et al., 2024; Veraksa et al., 2022, 2023). This trend exists against a backdrop of a general decline in the level of play activity, reduced free time for play, limited access to mixed-age peer groups, and the displacement of traditional play by digital (Veraksa et al., 2023; Dovolnova, 2023; Ngyah-Etchutambe, 2025; Nikolaeva et al., 2023; Smirnova, 2019). Taken together, these trends may limit opportunities for parent–child play, reducing the child’s initiative for parent–child play and its duration.

Since a child’s social situation of development is shaped by adults (Ryabkova and Mazmaniants, 2024; Smirnova, 2019; Vlasova et al., 2025), identifying family factors that are associated with the frequency and duration of parent–child play among preschool children attending preparatory groups in the final year before school entry becomes a relevant task. The study described in this article aimed to examine associations between parental play beliefs, family socio-demographic characteristics, parental retrospective childhood experiences, and indicators of parent–child play.

### 1.1 The role of the adult in parent–child play activity

Within cultural-historical and activity theory approaches, play is regarded as the leading activity of preschool age, through which the child symbolically masters the meanings of human activity (Vygotsky, 2017; Elkonin, 1978). Due to its social nature, play is shaped by upbringing and aspects of the child’s social situation of development (Elkonin, 1978). As Elkonin (1978, p. 85) noted, “The child’s world is, first and foremost, an adult as the most important part of the reality surrounding the child, a part of the adult world. It is only within the developing system of ‘child–adult’ relationships that the child enters the rest of the world”. Thus, the adult acts as a guide for the child into the world of human activity, work, and relationships, which is directly reflected in play activity—specifically, in the substantive content of the play role as an integral unit of play (Elkonin, 1978). The content of the mature form of play is the child’s reproduction of the system of social relations and adult activities in a generalized and abbreviated play form (Elkonin, 1978). Moreover, modeling systems of relationships and activities in play serves as an important means for adults to understand how the child perceives social reality and their place within it (Elkonin, 1978). From this perspective, parental play beliefs may reflect the extent to which parents support or constrain the realization of play as the leading activity in preschool age (Vygotsky, 2017; Elkonin, 1978).

### 1.2 Parental play beliefs and their associations with child development

The study of parental play beliefs is an important area of research, as these beliefs may be associated with the extent to which parents facilitate and support play activities at home (Metaferia et al., 2020b). For instance, parents who recognize the value and developmental role of play engage in parent–child play more frequently and for longer durations (Fogle and Mendez, 2006; Metaferia et al., 2020a). Empirical evidence indicates a positive association between parental Play Support and the development of children’s executive functions and social competence (Lin and Yawkey, 2013; Metaferia et al., 2020a,b; Plotnikova et al., 2024). This factor is also associated with higher quality of play in preschoolers, manifested in more developed collaborative play skills, imagination, emotional expressiveness, and concentration, whereas parental Academic Focus demonstrates a negative correlation (Fogle and Mendez, 2006; Li et al., 2022).

The study by LaForett and Mendez (2016) showed that Play Support was associated with parental education, level of depression, parenting self-efficacy, and parenting styles, with higher levels of depression and hostility negatively associated with this belief (LaForett and Mendez, 2016). Academic Focus was associated only with the child’s level of expressive language and the parents’ level of education (LaForett and Mendez, 2016). The results of the study by LaForett and Mendez (2016) are consistent with other research indicating that parental psychological well-being may be linked to parental attitudes, as well as to the child’s development and well-being (Apanovich and Serykh, 2024; Karpinski, 2025; Sobkin et al., 2024; Yakupova et al., 2024). In particular, active parental involvement in play is associated with reduced levels of parental stress and depressive symptoms, as well as with increased satisfaction with parent–child relationships (Berkule et al., 2014; Cates et al., 2016).

Parental play beliefs in relation to parental retrospective childhood experience remain an understudied area. In particular, limited attention has been paid to how these beliefs may vary depending on the characteristics of the home environment and experiences of residential mobility during childhood. Research indicates that the quality of both the psychological and physical home environment is associated with child development (Li et al., 2022). Specifically, a more favorable and structured home environment, along with parental support for play, has been associated with higher levels of play development in preschoolers (Li et al., 2022). Moreover, the home environment has been shown to mediate the relationship between family socioeconomic status (SES) and children’s play development. Accordingly, less favorable and less structured home environment, characterized by lower levels of Play Support, are associated with lower SES. In this context, it may be assumed that parental play beliefs are related to parents’ own experience of living in a less structured home environment during childhood. In addition, instability in the home environment may be linked to experiences of residential mobility, as such experiences may involve changes in family, social, and educational contexts (Anderson et al., 2014). Most empirical data indicate that the experience of moving can be associated with lower academic achievement and well-being, as well as problematic behavior in preschool and school age (Anderson et al., 2014; Henkens et al., 2024; Tønnessen et al., 2016; Vidal and Baxter, 2018). Moreover, Anderson et al. (2014) demonstrated that frequent moving is associated not only with general family instability but also with a decrease in the quality of the home learning environment, socioeconomic status, children’s and adolescents’ circle of friends, and changes in family composition. Research also suggests that moves in childhood and adolescence can be linked to outcomes in adulthood (Henkens et al., 2024; Tønnessen et al., 2016). It was found that children who moved during childhood were more likely to have lower educational attainment, lower income, and earlier parenthood in adulthood compared to children who did not move, with risks increasing with the number of moves (Henkens et al., 2024; Tønnessen et al., 2016). Thus, while associations between parental play beliefs and child development have been documented, less is known about how parental play beliefs are associated with parent-reported indicators of parent–child play, specifically the frequency of the child’s requests for parent–child play and the duration of such play, as well as about the associations of family socio-demographic characteristics and parental retrospective childhood experience with these beliefs.

### 1.3 The present study

This study aimed to examine associations between family factors and parent-reported indicators of parent–child play, such as the frequency of the child’s requests for parent–child play and the duration of parent–child play.

*H1:* Índicators of parent–child play are associated with parental play beliefs when controlling for family socio-demographic characteristics: parental age, education, employment status, and the number of children in the family.

*H2:* Parental Play Support belief mediates the associations between parental age and education and the parent-reported frequency of the child’s requests for parent–child play.

Additionally, the study pursued an exploratory aim, which was to investigate the connections between family factors—the experience of frequent moving and an unstructured home environment in the parental childhood, parental play beliefs, and family socio-demographic characteristics.

## 2 Materials and methods

### 2.1 Participants

The study involved 557 parents (M_age_ = 37.0, SD = 5.3) of typically developing children attending preparatory groups in preschool educational institutions, corresponding to the final year before school entry (M_age_ = 6.0, SD = 0.3; 46% girls). Of these, 374 children had siblings. Data on the parent’s relationship to the child were available for 543 participants: 511 respondents were mothers (94.1%), 28 were fathers (5.2%), and 4 were guardians (0.7%). The majority of respondents resided in Moscow (*n* = 433, 77.7%), followed by Kazan (*n* = 86, 15.4%) and Sochi (*n* = 38, 6.8%). Data on parental education were available for 546 respondents. Half of the parents had a bachelor’s degree (*n* = 273, 50.0%); 77 had a master’s degree (14.1%), 75 had a specialist degree (13.7%), 76 had secondary vocational education (13.9%), 32 had an academic degree (5.9%), and 13 had basic or complete general education (2.3%). Employment status was reported by 297 respondents: 162 were employed full-time (54.5%), 71 part-time (23.9%), and 64 were not employed (21.5%).

### 2.2 Procedure

Parents completed a paper-based questionnaire provided by the researchers.

### 2.3 Measures

The Parent Play Beliefs Scale (PPBS; Fogle and Mendez, 2006) was used to assess parental play beliefs. Participants were asked to rate 30 statements on a 5-point agreement scale. The instrument includes two subscales. The first subscale, “Play Support” (16 items; *α* = 0.90), reflects parents’ conviction about the importance of play, which brings not only enjoyment but also developmental benefits, with the parent as an active participant (e.g., “Play can help my child develop better thinking abilities”). The second subscale, “Academic Focus” (8 items; *α* = 0.70), expresses the parental belief in the priority of learning activities over play in the child’s development (e.g., “I do not think my child learns important skills by playing”).

A parent questionnaire was developed, consisting of two blocks. The first block contained questions about the family socio-demographic characteristics: age, gender, education, employment, and number of children. To retrospectively assess the structured home environment in the parental childhood, this block also included two questions about the experience of frequent moving and life organization: “In my childhood, my family moved frequently (changed residence)” and “When I was under 10 years old, there was rarely order and organization in my family.” Parents rated these from 1 (“Strongly Disagree”) to 5 (“Strongly Agree”). These items were developed for the exploratory assessment of aspects of chronic instability in the home environment (Anderson et al., 2014).

The second block included questions about parent–child play. To assess the frequency of the child’s requests for parent–child play, the question was: “How often in the past month has your child asked you to play with them?” Parents rated this on a scale from 1 to 6: 1 (“Not at all”), 2 (“Once this month”), 3 (“Two or three times this month”), 4 (“A couple of times a week”), 5 (“Almost every day”), 6 (“Every day”). To assess the duration of parent–child play, an open-ended question was used: “On average, how long does your play with your child last?” Parents were asked to indicate the time in minutes (full descriptive statistics and the histogram for this variable are provided in the Supplementary materials).

### 2.4 Data analysis

Data were analyzed using descriptive statistics, Spearman’s nonparametric correlation analysis, general linear models (GLM) with a Gaussian distribution and identity link function, and mediation analysis conducted in Jamovi (version 2.6.44.0). Due to deviations from normality of residuals (Shapiro–Wilk test, *p* < 0.001) and indications of heteroscedasticity (Breusch–Pagan test, *p* < 0.05), robust standard errors (HC3) were applied in general linear models. The significance of direct and indirect effects was evaluated using bias-corrected and accelerated (BCa) bootstrap confidence intervals based on 5,000 resamples. In addition, because the frequency of the child’s requests for parent–child play was assessed using an ordered 6-point item, an ordinal logistic regression model was fitted as a robustness analysis. The duration of parent–child play was analyzed using a winsorized duration variable, in which values above the 99th percentile were replaced with the 99th percentile value. In the present sample, the 99th percentile was 207 min.

## 3 Results

Correlation analysis revealed that the frequency of the child’s requests for parent–child play was positively correlated with Play Support (*ρ* = 0.248, *p* < 0.001), parental employment (*ρ* = 0.118, *p* = 0.045), and negatively correlated with the number of children (*ρ* = −0.217, *p* < 0.001), with effects ranging from small to moderate (Table 1). The duration of parent–child play was positively correlated with Play Support (*ρ* = 0.104, *p* = 0.017) and parental employment (*ρ* = 0.149, *p* = 0.012), and negatively correlated with parental age (*ρ* = −0.14, *p* = 0.001), with all small effects. No statistically significant correlations were found for either play indicator with Academic Focus or parental education (*p* > 0.05). The absence of associations between Academic Focus and indicators of parent–child play may be considered theoretically meaningful.

**Table 1:** Descriptive statistics and Spearman’s correlations between family factors and parent-reported indicators of parent–child play.

Variable	*N*	M	SD	Min	Max	1	2	3	4	5	6	7	8	9
Frequency of child’s requests	537	4.7	1.1	1	6	−								
Duration of parent–child play (min)	534	47.6	41.9	0	400	0.184^***^	−							
Play support	557	68.7	9.8	0	85	0.248^***^	0.104^*^	−						
Academic focus	557	16.6	4.6	0	40	−0.077	−0.026	−0.267^***^	−					
Parental age	537	37.0	5.3	24	66	−0.045	−0.140^**^	0.141^**^	−0.044	−				
Parental education	546	5.4	1.2	2	8	0.045	−0.034	0.114^**^	−0.051	0.130^**^	−			
Parental employment	297	2.3	0.8	1	3	0.118^*^	0.149^*^	0.011	0.043	−0.020	0.006	−		
Number of children	557	1.86	0.75	1	5	−0.217^***^	−0.069	0.041	−0.034	0.148^***^	−0.032	−0.319^***^	−	
Frequent moving (parental childhood)	541	1.6	1.01	1	5	0.057	0.042	−0.105^*^	0.080	−0.053	−0.021	−0.130^*^	0.036	−
Unstructured home env. (parental childhood)	540	1.8	1.07	1	5	−0.051	−0.008	−0.014	0.094^*^	−0.027	−0.123^**^	−0.080	0.057	0.224^***^

^*^p < 0.05, ^**^p < 0.01, ^***^p < 0.001. Correlation analyses were exploratory; *p*-values were not adjusted for multiple comparisons.

Additionally, within the exploratory aim, it was found that Play Support was positively associated with parental age (*ρ* = 0.141, *p* = 0.001) and education (*ρ* = 0.114, *p* = 0.008), and negatively associated with the experience of frequent moving in the parental childhood (*ρ* = −0.105, *p* = 0.015). Academic Focus was positively associated with the experience of an unstructured home environment in the parental childhood (*ρ* = 0.094, *p* = 0.03). The experience of frequent moving was positively correlated with an unstructured home environment (*ρ* = 0.224, *p* < 0.001) and negatively correlated with parental employment (*ρ* = −0.130, *p* = 0.027). The experience of an unstructured home environment was negatively correlated with the parental education level (*ρ* = −0.123, *p* = 0.004). All observed effects were small.

### 3.1 Associations of parental play beliefs and family socio-demographic characteristics with parent–child play indicators

**Figure 1:**
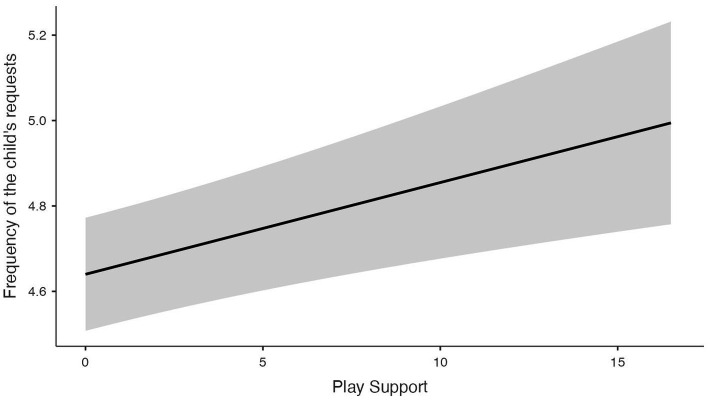
Graphs of the GLM showing associations between the frequency of the child’s requests for parent–child play and parental play support belief.

To test H1, the frequency of the child’s requests was included as the dependent variable and Play Support, number of children, and parental employment as independent variables. This model was significant (*R*^2^ = 0.084; *F* = 6.53; *p* < 0.001; *N* = 290), indicating a modest proportion of explained variance given the characteristics of the dependent variable. Play Support was positively associated with the frequency of the child’s requests [SE = 0.01; 95% CI (0.008, 0.043); *β* = 0.2; *p* = 0.03] (Figure 1), whereas the number of children was negatively associated with the frequency of the child’s requests [SE = 0.08; 95% CI (−0.5, −0.159); *β* = −0.2; *p* < 0.001], with both associations showing small to moderate effect sizes. Employment status was not significantly associated with the frequency of the child’s requests (*p* > 0.05).

As an additional robustness analysis, an ordinal logistic regression model was fitted because the frequency of the child’s requests was an ordered outcome. This model was significant (*χ*^2^ = 29.5; df = 4; *p* < 0.001; *N* = 290), indicating modest explanatory capacity (McFadden’s *R*^2^ = 0.036). Likelihood-ratio omnibus tests showed significant effects of Play Support (*χ*^2^ = 12.30; df = 1; *p* < 0.001) and the number of children (*χ*^2^ = 14.50; df = 1; *p* < 0.001). The effect of parental employment was not statistically significant (*χ*^2^ = 0.56; df = 2; *p* = 0.755). Higher Play Support was associated with greater odds of the child being rated in a higher request-frequency category [*b* = 0.042; SE = 0.013; OR = 1.043; 95% CI (1.018, 1.069); *p* < 0.001]. Conversely, a larger number of children was associated with lower odds of the child being rated in a higher request-frequency category [*b* = −0.590; SE = 0.156; OR = 0.554; 95% CI (0.407, 0.751); *p* < 0.001].

**Figure 2:**
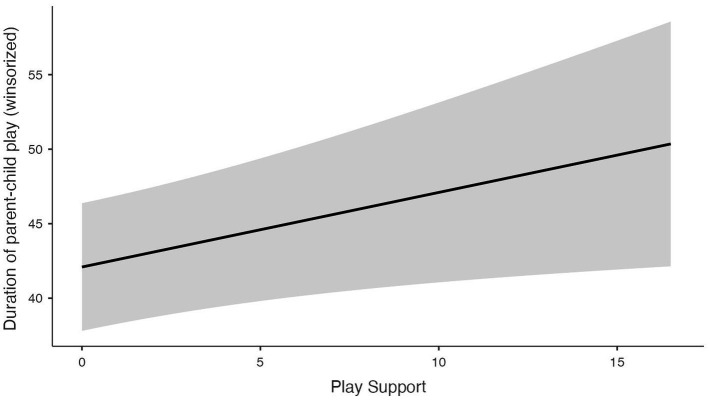
Graphs of the GLM showing associations between the duration of parent–child play and parental play support belief.

The duration of parent–child play showed a right-skewed distribution (M = 47.6; SD = 41.9; Mdn = 30.0; IQR = 30.0; range = 0–400). To assess the robustness of the findings to high-duration values, a sensitivity analysis was conducted using a winsorized duration variable. The 99th percentile was 207 min and was used as the winsorization threshold (see Supplementary materials). The model with the winsorized duration variable as the dependent variable and Play Support, parental age, and employment as independent variables was significant (*R*^2^ = 0.083; *F* = 6.06*; p* < 0.001; *N* = 272), accounting for a modest proportion of variance in the dependent variable. Parental age was negatively associated with the duration of parent–child play [SE = 0.380, 95% CI (−2.190, −0.670), *β* = −0.196, *p* < 0.001], showing a small to moderate effect size. Play Support was positively associated with the duration of parent–child play in the winsorized model [SE = 0.236, 95% CI (0.108, 1.032), *β* = 0.137, *p* = 0.035], showing a small effect size. Employment status was also significantly associated with play duration (*p* = 0.004). *Post-hoc* Tukey tests showed that parents employed full-time reported longer parent–child play duration than non-working parents, with a mean difference of 14.82 min (*p* = 0.003). No significant differences were found between part-time and full-time employed parents or between part-time employed and non-working parents (Figure 2).

### 3.2 Parental play beliefs as mediators of the associations between family socio-demographic characteristics and indicators of parent–child play

**Figure 3:**
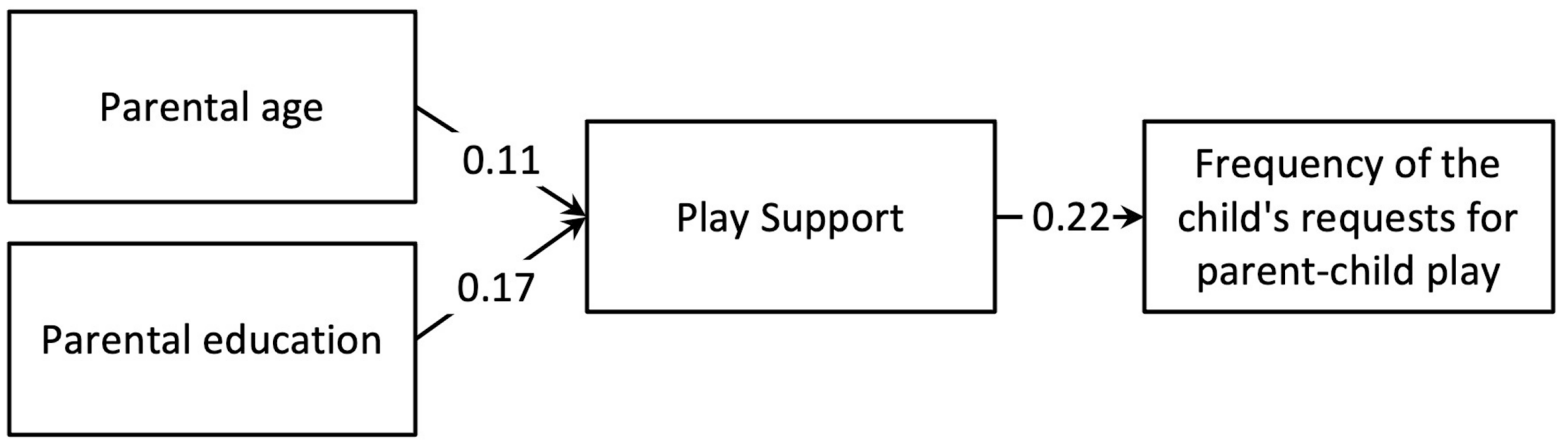
Play support as a mediator in the relationship between parental age and education and the frequency of the child’s requests for parent–child play. Standardized coefficients (*β*) are presented.

To test H2, the frequency of the child’s requests for parent–child play was included as the dependent variable, Play Support as the mediator, and parental age and education as independent variables (*N* = 516). Significant positive indirect effects were found for both parental age [SE = 0.002; 95% CI (0.001, 0.01); *β* = 0.024; *p* = 0.026] and education [SE = 0.01; 95% CI (0.016, 0.06); *β* = 0.04; *p* = 0.002], both reflecting small effect sizes, in the absence of significant direct and total effects (*p* > 0.05). Path analysis supported this pattern: both parental age [SE = 0.08; 95% CI (0.04, 0.34); *β* = 0.11; *p* = 0.014] and parental education [SE = 0.4; 95% CI (0.6, 2.41); *β* = 0.2; *p* < 0.001] were positively associated with the level of Play Support, with small and small to moderate effect sizes, respectively. Play Support, in turn, was positively associated with the frequency of the child’s requests for parent–child play [SE = 0.004; 95% CI (0.013, 0.04); *β* = 0.2; *p* < 0.001] (Figure 3).

## 4 Discussion

This article presents the results of a study examining family factors associated with the frequency with which children attending preparatory preschool groups in Russia, corresponding to the final year before school entry, ask their parents to play together, as well as the duration of this parent–child play. The main finding was that parental Play Support was positively associated with the frequency of the child’s requests for parent–child play. Another result was that Academic Focus was not associated with either the frequency of requests or the duration of play. The cumulative consideration of these relationships suggests that parents who recognize the developmental potential of play may participate more often in parent–child play, while children in such family contexts may show more frequent initiative toward parent–child play. Furthermore, the finding that Academic Focus was not associated with play indicators underscores the enduring status of play as the leading activity in preschool age, including in the final year before school entry. These data suggest that a child’s interest in play may remain strong and relatively independent of parental beliefs oriented toward shifting the leading activity.

H1 was partially supported. Among parental play beliefs, Play Support was positively associated with both parent-reported indicators of parent–child play: the frequency of the child’s requests for parent–child play and the duration of such play. Academic Focus, in contrast, was not significantly associated with either play indicator. Among socio-demographic characteristics, the number of children in the family was negatively associated with the frequency of the child’s requests, whereas parental employment was not significantly associated with this indicator. For the duration of parent–child play, parental age was negatively associated with play duration, and employment status was also significantly associated with duration: parents working full-time reported longer parent–child play compared to non-working parents.

Thus, parents who more strongly endorsed the developmental value of play tended to report more frequent requests from the child for parent–child play and longer play duration. This result is consistent with previous research linking parental play beliefs to parents’ involvement in play (Fogle and Mendez, 2006; LaForett and Mendez, 2016; Li et al., 2022; Metaferia et al., 2020b), and extends it by showing that Play Support is also associated with parent-reported child initiative toward parent–child play. The significance of this finding is heightened by the modern context of declining levels of play activity and diminishing opportunities for play among children in the final year before school entry. In this situation, a decline in child initiative may reduce opportunities for the child to engage in play as the leading activity of preschool age, which is considered a primary source of development during this sensitive period (Plotnikova et al., 2024; Smirnova, 2019). Therefore, parental beliefs in the developmental value of traditional play and parental participation in such play may be associated with the maintenance of children’s tendency to seek parent–child play, which may be relevant to the development of more mature forms of play. At the same time, it is important to note that the observed effects were small in magnitude. This may partly reflect the fact that all variables were assessed using parent reports, which may introduce common-method variance and limit the precision with which behavioral aspects of parent–child play can be captured. Future research should therefore include observational measures of parent–child play and more direct behavioral indicators of children’s play initiative and parental involvement, which may allow for a more precise assessment of these associations.

Furthermore, the lack of association between Academic Focus and play indicators may suggest that during preschool age, including in the final year before school entry, children’s orientation toward play remains relatively independent of parental beliefs emphasizing formal learning (Elkonin, 1978; Novlyanskaya, 2025; Vygotsky, 2017). Thus, play retains its status not only as the child’s leading activity but also as an important form of meaningful, developmental interaction with an adult. This interpretation is further supported by findings from other cultural contexts. For example, in a sample of Ethiopian preschoolers, children’s frequency of engagement in pretend play, motor play, solitary play, and peer play was not associated with parental belief in Academic Focus, which was instead related only to the frequency of academics-related activities (Metaferia et al., 2020b). A similar finding was observed in a sample of Hungarian preschoolers (Metaferia et al., 2020a). This pattern may suggest that an emphasis on academic activities does not necessarily extend to children’s play behavior across different cultural contexts. In the Russian context, the absence of this association may reflect the persistence of cultural values that prioritize play despite increasing academic pressures.

The results also showed that a larger number of children in the family was associated with less frequent parent-reported requests for play with the parent. This association may be related to the fact that siblings provide a natural context for peer interaction, within which children may more often engage in joint play (Smirnova, 2019). Unlike the asymmetrical interaction with an adult, communication with siblings can be characterized by mutual play needs, spontaneous initiative, and a position of equal partners. The availability of siblings as potential play partners at home may be associated with a lower need to initiate play specifically with a parent and with fewer situational barriers to play with other children.

It was also revealed that parental employment was associated with the duration of parent–child play. Specifically, parents working full-time reported longer play interactions with their children compared to non-working parents. An explanation for this may be a mutual need for emotionally rich contact: parent–child play may become a way to reconnect after separation during the day (Lisina, 1974; Berkule et al., 2014; Cates et al., 2016; LaForett and Mendez, 2016). However, this interpretation is *post hoc* and should be treated with caution, requiring further empirical investigation.

Parental age was negatively associated with the duration of parent–child play, suggesting that older parents reported shorter play interactions with their children. It is possible that older parents may act more as facilitators of the play process, initiating the child’s play with less subsequent direct involvement. Research has shown that the purposeful inclusion of an adult as an organizer or facilitator of sociodramatic play is associated with the development of the child’s executive functions more strongly than free play without adult participation (Veresov et al., 2021). These data suggest that for child development, the qualitative characteristics of play—such as effective initiation and the organization of an environment conducive to its subsequent unfolding—may be more significant than the quantitative indicator of duration.

The H2 was supported. The frequency of the child’s requests was significantly positively associated with Play Support, which, in turn, was positively associated with parental age and education. Notably, no direct or total effects of parental age and education on the frequency of requests were detected. This pattern indicates indirect-only mediation through Play Support. However, the observed indirect effects were small in magnitude and should therefore be interpreted cautiously. This suggests that older and more educated parents tend to report a stronger pro-play stance, which is associated with more frequent child requests for parent–child play. One possible interpretation is that the life experience and knowledge of child development characteristic of older and more educated parents may be associated with a stronger recognition of the importance of play, which, in turn, may be linked to more frequent child requests for parent–child play. This finding is consistent with the study by LaForett and Mendez (2016), which also found a link between education level and parental play beliefs. The absence of a link between parents’ education and Academic Focus in the present study might be explained by cultural context: within the Russian educational tradition, the value of learning persists regardless of the level of parents’ formal education (Kortava, 2024).

Within the exploratory aim, correlation analysis revealed two significant associations. Specifically, Academic Focus was positively associated with the experience of an unstructured home environment in the parent’s childhood, whereas Play Support was negatively associated with the experience of frequent moving. It can be assumed that the Academic Focus belief in parents who experienced life disorganization in childhood may be linked to compensatory strategies aimed at creating a more structured educational environment for their own children, possibly as a reaction to disruptions in their own childhood learning (Anderson et al., 2014; Henkens et al., 2024; Tønnessen et al., 2016). In turn, the negative association between frequent moving and Play Support may reflect more limited opportunities for stable play experiences in childhood. Taken together, these exploratory findings suggest that parents’ retrospective childhood experiences may be linked to their current play beliefs. However, given that the retrospective childhood environment was assessed using single-item measures, these associations should be interpreted cautiously. Future research should examine these links using more reliable and valid measures of retrospective childhood environment (Frankenburg and Coons, 1986), which may help clarify the role of parents’ own childhood experiences in the formation of parental play beliefs.

Thus, the study suggests that parental understanding of the developmental value of play may be an important correlate of parent-reported indicators of parent–child play. Parental Play Support was positively associated with parents’ reports of how often children attending preparatory preschool groups requested joint play and with the reported duration of parent–child play. Play Support also showed an indirect-only mediation pattern in the associations of parental age and education with the frequency of the child’s requests. Analysis of other family factors revealed that having siblings was associated with less frequent child requests for play with the parent; parental employment was positively related to play duration and older parental age was associated with shorter play duration. Within the exploratory aim of the study, parental retrospective childhood experiences were also associated with parental play beliefs. Specifically, an unstructured home environment in the parent’s own childhood was positively associated with Academic Focus, whereas the experience of frequent moving was negatively associated with Play Support.

## 5 Conclusion

This article presented the results of a study on associations between family factors and parent-reported indicators of parent–child play among children attending preparatory preschool groups in Russia during the final year before school entry. Parental Play Support was positively associated with the frequency of the child’s requests for parent–child play and the duration of play. This belief showed an indirect-only mediation pattern in the associations of parental age and education with the frequency of the child’s requests. Parental Academic Focus was not associated with either the frequency of requests or the duration of play. Socio-demographic characteristics were also associated with indicators of parent–child play: a larger number of children in the family was negatively associated with the frequency of requests, full-time parental employment was associated with longer play duration compared to non-employment, and parental age was negatively associated with play duration. Within the exploratory aim of the study, parental retrospective childhood experiences were associated with parental play beliefs. Specifically, the experience of an unstructured home environment in the parent’s childhood was positively associated with Academic Focus, whereas the experience of frequent moving was negatively associated with Play Support.

The described study has several limitations. First, all variables were assessed using parent reports, including parental play beliefs, family socio-demographic characteristics, parental retrospective childhood experiences, and indicators of parent–child play. This may increase the risk of common-method variance, as the observed associations may be partly influenced by shared informant and method effects. In addition, parent-reported data may be subject to social desirability and recall biases. Second, the cross-sectional design of the study does not allow conclusions about the directionality or causality of the observed associations. Third, the respondent sample consisted predominantly of mothers (94%) and included a large proportion of families residing in Moscow (78%). This limits the generalizability of the findings to fathers, other caregivers, and families living in other regional and socio-cultural contexts. Fourth, the exploratory assessment of parental retrospective childhood experiences was based on two single-item measures. Although these items allowed us to address specific aspects of childhood home instability relevant to the exploratory aim of the study, they may have limited reliability and validity. Therefore, the findings concerning parental retrospective childhood experiences should be interpreted with caution. Future research should use more reliable and valid instruments for assessing retrospective childhood environment, such as retrospective adaptations of the HOME inventory (Frankenburg and Coons, 1986). Fifth, no *a priori* sample-size justification or power analysis was conducted before data collection, including for the mediation analyses. The final sample size depended on the voluntary response rate among parents recruited through preschool educational institutions. Therefore, the mediation analyses, particularly the small indirect effects, should be interpreted with caution.

## Data Availability

The raw data supporting the conclusions of this article will be made available by the authors, without undue reservation.

## References

[ref1] Anderson S. Leventhal T. Newman S. Dupéré V. (2014). Residential mobility among children: a framework for child and family policy. City 16, 5–36.

[ref2] Apanovich T. M. Serykh A. B. (2024). Emotional burnout symptoms in mothers of primary school children: role of intensive parenting attitudes and parenting styles. Russ. Psychol. J. 21, 22–34. doi: 10.21702/rpj.2024.2.2

[ref3] Berkule S. B. Cates C. B. Dreyer B. P. Huberman H. S. Arevalo J. Burtchen N. . (2014). Reducing maternal depressive symptoms through promotion of parenting in pediatric primary care. Clin. Pediatr. 53, 460–469. doi: 10.1177/0009922814528033, 24707022 PMC4435690

[ref4] Cates C. B. Weisleder A. Dreyer B. P. Johnson S. B. Vlahovicova K. Ledesma J. . (2016). Leveraging healthcare to promote responsive parenting: impacts of the video interaction project on parenting stress. J. Child Fam. Stud. 25, 827–835. doi: 10.1007/s10826-015-0267-7, 27134514 PMC4847426

[ref5] Dovolnova I. V. (2023). The specifics of the manifestations of initiative by boys and girls of preschool and primary school age in the socio-cultural environment. Preschool Educ. Today 16, 54–64. doi: 10.24412/2782-4519-2023-1115-54-64

[ref6] Elkonin D. B. (1978). Psychology of Play. Moscow: Pedagogika.

[ref7] Fogle L. M. Mendez J. L. (2006). Assessing the play beliefs of African American mothers with preschool children. Early Child Res. Q. 21, 507–518. doi: 10.1016/j.ecresq.2006.08.002

[ref8] Frankenburg W. K. Coons C. E. (1986). Home screening questionnaire: its validity in assessing home environment. J. Pediatr. 108, 624–626. doi: 10.1016/s0022-3476(86)80853-8 3958839

[ref9] Henkens J. H. D. Stevens G. W. J. M. de Valk H. A. G. (2024). The relation between residential mobility and internalizing and externalizing problems in adolescence: the role of subjective moving experience, gender, and friendship quality. J. Youth Adolesc. 53, 2234–2250. doi: 10.1007/s10964-024-02014-6, 38789875 PMC11413126

[ref10] Karpinski K. V. (2025). Children as agents of adults’ psychological well-being. Psychol. Educ. 7, 40–58. doi: 10.33910/2686-9527-2025-7-1-40-58

[ref11] Kortava T. V. (2024). Power of word in the education of a person and a citizen of the fatherland. Lomonosov Pedagog. Educ. J. 22, 54–71. doi: 10.55959/LPEJ-24-03

[ref12] LaForett D. Mendez J. L. (2016). Play beliefs and responsive parenting among low-income mothers of preschoolers in the United States. Early Child Dev. Care 187, 1359–1371. doi: 10.1080/03004430.2016.1169180, 37339054

[ref13] Li S. Sun J. Dong J. (2022). Family socio-economic status and children’s play behaviors: the mediating role of home environment. Children 9:1385. doi: 10.3390/children9091385, 36138694 PMC9497565

[ref14] Lin Y.-C. Yawkey T. (2013). Does play matter to parents? Taiwanese parents’ perceptions of child’s play. Education 134, 244–254.

[ref15] Lisina M. I. (1974). Communication and its Influence on the Mental Development of a Preschool Child. Collection of Scientific Works. Moscow.

[ref16] Metaferia B. K. Futo J. Drew R. Takacs Z. K. (2020a). Parents’ beliefs about play and the purpose of preschool education, preschoolers’ home activity and executive functions. Front. Psychol. 11:1104. doi: 10.3389/fpsyg.2020.01104, 32670139 PMC7326145

[ref17] Metaferia B. K. Takacs Z. K. Futo J. (2020b). The relationship between parental play beliefs, preschoolers’ home experience, and executive functions: an exploratory study in Ethiopia. Front. Psychol. 11:624. doi: 10.3389/fpsyg.2020.00624, 32373015 PMC7185235

[ref18] Ngyah-Etchutambe I. B. (2025). Children’s engagement in games and the development of 21st century competencies: a comparison of indigenous and digital games in late childhood. New Ideas Child Educ. Psychol. 5, 3–21. doi: 10.11621/nicep.2025.0501

[ref19] Nikolaeva E. I. Kalabina I. A. Progackaya T. K. Ivanova E. V. (2023). Ground rules for preschooler exposure to the digital environment: a review of studies. Psychol. Russ. 16, 37–54. doi: 10.11621/pir.2023.0403, 38162805 PMC10755961

[ref20] Novlyanskaya Z. N. (2025). Transition from play to learning in the course of literary reading in the system of developmental education by D.B. Elkonin and V.V. Davydov. Mosc. Univ. Psychol. Bull. 48, 294–317. doi: 10.11621/LPJ-25-34

[ref21] Oshchepkova E. S. Sukhikh V. L. Shatskaya A. N. (2023). The influence of various types of play on the development of coherent monologue speech in children aged 5-6 years. RUDN J. Psychol. Pedagog. 20, 464–481. doi: 10.22363/2313-1683-2023-20-3-464-481

[ref22] Plotnikova V. A. Gavrilova M. N. Fedorova A. V. (2024). Trends in the play of modern children and their connection with mental development: empirical study. Perspect. Sci. Educ. 67, 470–489. doi: 10.32744/pse.2024.1.26

[ref23] Ryabkova I. A. Mazmaniants M. G. (2024). Teachers’ and psychologists’ attitude to a toy: the historical dynamics of value orientations in Russia. Mosc. Univ. Psychol. Bull. 47, 316–350. doi: 10.11621/LPJ-24-26

[ref24] Smirnova E. O. (2019). Specificity of modern preschool childhood. Natl. Psychol. J. 34, 25–32. doi: 10.11621/npj.2019.0205

[ref25] Sobkin V. Ryabkova I. A. Sozinova I. V. (2024). A preschooler’s emotional well-being within a family (based on psychological analysis of children’s drawings). New Ideas Child Educ. Psychol. 4, 40–66. doi: 10.11621/nicep.2024.0403

[ref26] Tønnessen M. Telle K. Syse A. (2016). Childhood residential mobility and long-term outcomes. Acta Sociol. 59, 113–129. doi: 10.1177/0001699316628614

[ref27] Veraksa A. N. Kurilenko V. B. Novikova I. A. (2023). Phenomenology of childhood in modern contexts. RUDN J. Psychol. Pedagog. 20, 419–430. doi: 10.22363/2313-1683-2023-20-3-419-430, 33661674

[ref28] Veraksa A. Sukhikh V. Veresov N. Almazova O. (2022). Which play is better? Different play types and development of executive functions in early childhood. Int. J. Early Years Educ. 30, 560–576. doi: 10.1080/09669760.2022.2091979

[ref29] Veresov N. Veraksa A. Gavrilova M. Sukhikh V. (2021). Do children need adult support during sociodramatic play to develop executive functions? Experimental evidence. Front. Psychol. 12:779023. doi: 10.3389/fpsyg.2021.779023, 34938243 PMC8685249

[ref30] Vidal S. Baxter J. (2018). Residential relocations and academic performance of Australian children: a longitudinal analysis. Longit. Life Course Stud. 9, 133–156. doi: 10.14301/llcs.v9i2.435

[ref31] Vlasova V. K. Zakirova V. G. Zharkovskaya I. O. (2025). Modern animation and the younger schoolchild: didactic possibilities and diagnostic potential. Educ. Self Dev. 20, 103–117. doi: 10.26907/esd.20.1.08

[ref32] Vygotsky L. S. (2017). Play and its role in the mental development of the child. Almanac Inst. Correct. Pedagogy 28, 1–33.

[ref33] Yakupova V. A. Anikeeva M. A. Suarez A. D. (2024). Maternal depression and development of child executive functions: foreign literature review. Theor. Exp. Psychol. 17, 123–142. doi: 10.11621/TEP-24-07

