# Supplementary material for: Parental play beliefs as a factor in the frequency of preschoolers’ requests for parent–child play and its duration
Source: Front Psychol. 2026 May 21;17:1749925. doi: 10.3389/fpsyg.2026.1749925 (PMC13233385; doi:10.3389/fpsyg.2026.1749925)
Supplement: Supplementary file 1 [file Data_Sheet_1.pdf]

Appendix: Parent Questionnaire on Parent-Child Play and Parental Retrospective Childhood Experience

1. How often in the past month has your child asked you to play with them? Please circle the appropriate option.

- Not at all
- Once this month
- Two or three times this month
- A couple of times a week
- Almost every day
- Every day

2. On average, how long does your play with your child last? Please indicate in minutes: ____________________

For each statement, please indicate the number that best reflects your level of agreement:

3. In my childhood, my family moved frequently (changed residence).

1 - Strongly disagree

2 – Disagree

3 – Neither agree nor disagree

4 – Agree

5 – Strongly agree

4. When I was under 10 years old, there was rarely order and organization in my family.

1 - Strongly disagree

2 – Disagree

3 – Neither agree nor disagree

4 – Agree

5 – Strongly agree
