# Supplementary material for: Parental play beliefs as a factor in the frequency of preschoolers’ requests for parent–child play and its duration
Source: Front Psychol. 2026 May 21;17:1749925. doi: 10.3389/fpsyg.2026.1749925 (PMC13233385; doi:10.3389/fpsyg.2026.1749925)
Supplement: Supplementary file 2 [file Data_Sheet_2.pdf]

Table S1

Descriptive statistics for the parent-reported duration of parent-child play

Descriptive statistic	Duration of parent-child play	Duration of parent-child play (winsorized)
N	534	534
Missing	23	23
Mean	47.6	46.5
Median	30.0	30.0
Standard deviation	41.9	35.4
Variance	1754	1255
Minimum	0	0
Maximum	400	207
25th percentile	30.0	30.0
50th percentile	30.0	30.0
75th percentile	60.0	60.0
Interquartile range (IQR)	30.0	30.0
95th percentile	120	120
99th percentile	207	205
