# Supplementary figures and images for: Parental play beliefs as a factor in the frequency of preschoolers’ requests for parent–child play and its duration
Source: Front Psychol. 2026 May 21;17:1749925. doi: 10.3389/fpsyg.2026.1749925 (PMC13233385; doi:10.3389/fpsyg.2026.1749925)

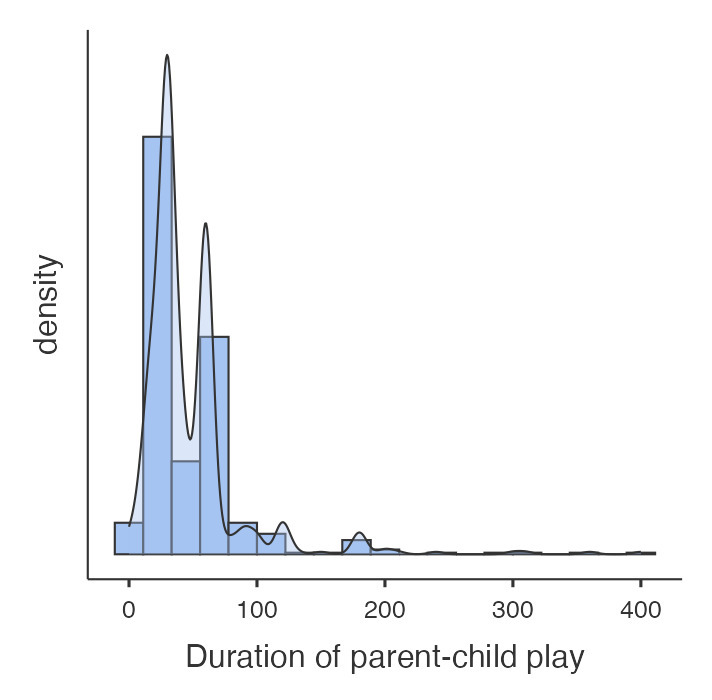

Supplement: Supplementary file 3 [file Image_1.jpeg]

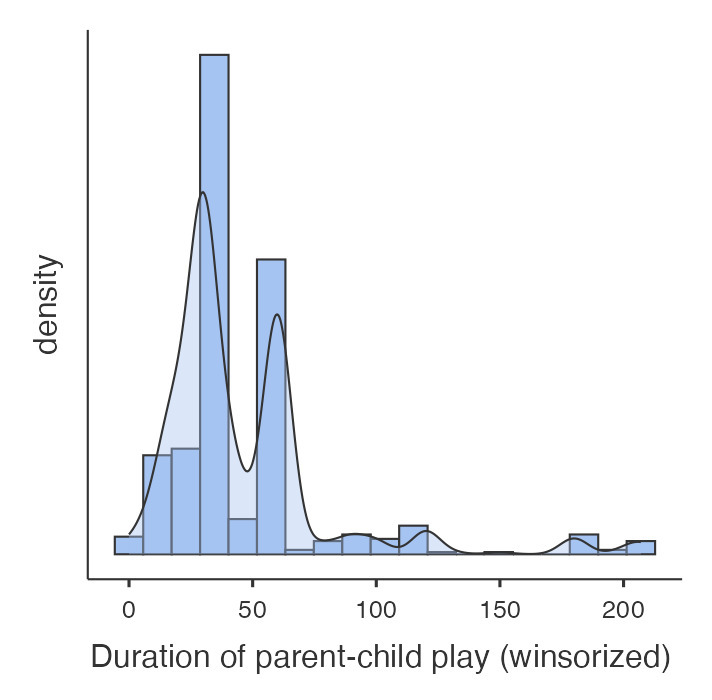

Supplement: Supplementary file 4 [file Image_2.jpeg]
